# Fish assemblage changes over half a century in the Yellow River, China

**DOI:** 10.1002/ece3.3890

**Published:** 2018-03-30

**Authors:** Jia Yan Xie, Wen Jia Tang, Yu Hui Yang

**Affiliations:** ^1^ School of Biology and Pharmaceutical Engineering Wuhan Polytechnic University Wuhan, Hubei China; ^2^ Qinghai Eco‐environment Remote Sensing Monitoring Center Xining China

**Keywords:** anthropogenic influences, dams, fish fauna, species invasions

## Abstract

Riverine environments have been threatened by anthropogenic perturbations worldwide, whereby their fish assemblages have been modified by habitat changes and nonendemic species invasions. We assessed changes in fish assemblages by comparing the species presence in historical and contemporary fish data in the Yellow River from 1965 to 2015. The temporal change in species assemblages was found with increased nonendemic species and fewer natives. Fish species richness of the river declined 35.4% over the past fifty years. Moreover, the decreased mean Bray–Curtis dissimilarity among reaches suggested that the fish assemblages of different reaches in the Yellow River were becoming more similar over time. However, temporal patterns of fish assemblages varied among reaches. In the upper Yellow River, higher species richness and more invasive species were found than those in the historical record, while the lower reaches experienced significant species loss. Dam constructions, exotic fish invasions, and flow reductions played the vital role in structuring the temporal fish assemblages in the Yellow River. It is suggested that river basins which experienced different types and levels of stressors by anthropogenic perturbations can produce varied effects on their temporal trends of species assemblages.

## 1 INTRODUCTION

With the rapid economic growth and urban expansion, aquatic environments have been threatened by biological invasions and anthropogenic perturbations which result from hydropower station, irrigation and industrial usage (Argent & Carline, 2004; Graf, 2006). Resulting changes to those riverine ecosystems could form a new water ecological environment which could affect the fish fauna composition by modifying the resistance to the disturbance or invasion (Hooper et al., 2005), and by altering the survival and reproduction of aquatic organisms (Miller, Williams, & Williams, 1989). Anthropogenic disturbances (e.g., habitat alteration by the construction of dams) have been an important driver of current and anticipated alterations in fish species assemblages, as well as biotic homogenization in aquatic ecosystems (Głowacki & Penczak, 2013; Liermann, Nilsson, Robertson, & Ng, 2012; Poff, Olden, Merritt, & Pepin, 2007; Rahel, 2000). When natural flow regimes, which could effectively prevent the establishment of alien fishes, are stabilized by dams, non‐natives dominate and displace natives through competition and predation. Meanwhile, introductions of non‐natives and extirpations of native fishes in rivers can accelerate the similarity among biotas, thus causing their homogenization (McKinney & Lockwood, 1999; Olden & Rooney, 2006). Changes in species assemblages have significantly altered ecosystem properties (Hooper et al., 2005). Furthermore, biotic homogenization can result in biotas losing biological distinctiveness (Olden & Rooney, 2006) and further increase the vulnerability of the ecosystem by simplifying trophic structures (Olden, Poff, Douglas, Douglas, & Fausch, 2004). However, such extensive habitat alteration by humans in the riverine ecosystem has been greatly conducting on the global scales (Poff et al., 2007)‎. Nearly half of worldwide large river systems are influenced by dams (Lehner et al., 2011)‎. Although constructions of hydropower stations could generate huge economic benefits, they also cause considerable ecological damages for freshwater ecosystems (Mims & Olden, 2013). Nevertheless, constructions of dams in the future, particularly in developing nations, are inevitable to satisfy the demand for human population growth and electricity needs (Palmer et al., 2008). Therefore, monitoring changes in fish assemblages after anthropogenic disturbances is effective for assisting planners to elucidate the trade‐offs associated with each decision in the ecologically sustainable river management (Poff & Zimmerman, 2010)‎.

The Yellow River has been intensively modified by anthropogenic habitat alterations and introductions of non‐native species (CTFRYR, 1986)‎. As the earliest hydraulic resource development base from the beginning of the 1960s in China, 32 large and medium‐size dams in the main stem and hundreds of smaller ones in the tributaries have been constructed or impounded in the Yellow River (Kou, Niu, Huang, Pang, & Yang, 2009; The Yellow River Conservancy Commission of Ministry of Water Resources Water, 2012; Yang, 1997)‎. There is abundant aquatic biodiversity and a high degree of endemism of fish assemblages in the river (Chen, Chen, & Liu, 1996; He & Chen, 2006; Li, 1965). However, the ichthyofauna and stream ecological communities in the Yellow River are confronted with the threats of river fragmentation (e.g., cascading impoundments), introductions of exotic species, and overexploitation (Tang & He, 2013)‎. In China, however, the water conservancy project is shifting the focus to the rivers in Southwest China (e.g., Ya‐lung River, Lancang River), the primary existing natural habitats for endemic fish species. It is critical to mitigate future impacts of dams on the riverine ecosystem. Therefore, as the earliest hydraulic resource development base, the Yellow River can offer an ideal location to evaluate the influence of changes caused by anthropogenic habitat alterations and introductions of alien fishes on fish assemblages, which may provide implications for the water conservancy development in the future.

In this study, we examined temporal patterns in fish community structure among five reaches in the Yellow River by comparing species presences in historical surveys with recent data from 1965 to 2015. We hypothesized that there are temporal alterations of the fish assemblages in the Yellow River from 1965 to 2015. We also hypothesized a reduction in contemporary fish species richness with increased nonendemic fishes and decreased natives, and an increase in taxonomic similarity between different reaches in the Yellow River over time. Additionally, we discussed if environmental variables including dam constructions, exotic fish invasions, and flow reductions may be responsible for shaping current fish assemblages in the Yellow River.

## 2 MATERIALS AND METHODS

### 2.1 Study area

**Figure 1:**
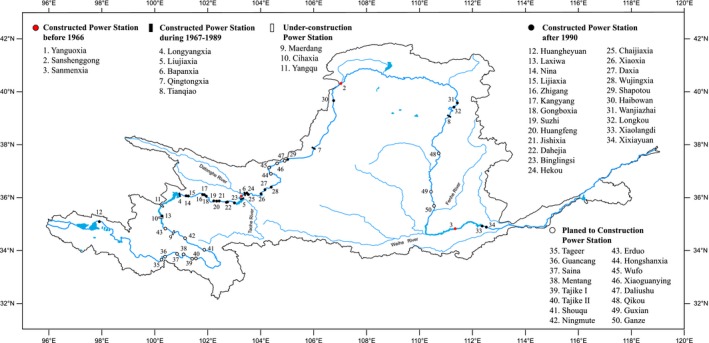
Location of power stations in the Yellow River, China. Different geometries denote the periods when dams were built, red closed circles: constructed power stations before 1966; black closed squares: constructed power stations during 1967–1989; black closed circles: constructed power stations after 1990; gray closed squares: under‐construction power station; open circles: planed to construction power station

The 5,464‐km‐long Yellow River (Figure 1), the second‐longest river in China, flows from west to east through nine provinces, and empties to the Bohai Gulf, China (CTFRYR, 1986). The Yellow River originates in the Bayan Har Mountains at 4,800 in western China and runs through the valley section stretches. Steep cliffs line both sides of the upper river with a high‐elevation drop, resulting in upper streams with the increased turbulent flow and higher flow velocities. This topography renders upper segments the best location for hydroelectric plant constructions. The river next enters a section of the vast alluvial plains, the Yinchuan Plain and Hetao Plain. In this section, the areas along the river are most deserts and grasslands. Then, the middle reaches of the Yellow River pass through the Loess Plateau, where a large amount of mud and sand discharged into the river results in the Yellow River, the most sediment‐laden river in the world. The river then flows easterly into the Bohai Gulf (CTFRYR, 1986). In the middle and lower reaches of the river, dramatic reductions in flow are associated with the increased social and economic consumption of water accompanied with the increasing population urbanization extending from west to east (CTFRYR, 1986; Liu & Cheng, 2000). Therefore, in order to meet the social and economic demand, the Yellow River becomes the earliest hydraulic resource development base in China. There are 32 large and medium‐size dams in the main stem of the Yellow River at present (Figure 1), where three dams have been built before 1966 (Yang, 1997; Yang & Xin, 1998). Five dams were built during 1967–1989, and 24 dams were constructed during 1990–2015 (Ding & Zhang, 2014; Kou et al., 2009; Li, 2005; Sun, Zhuang, Zhang, & Zheng, 2012; Zhan, Zhang, & Qing, 2009; Zhu, 1999). About three‐fourth of all dams were constructed in the upper and middle reaches of the Yellow River since 1961. And now, three dams are being constructed and sixteen power stations will be built in the Yellow River (Figure 1).

### 2.2 Data collection

**Figure 2:**
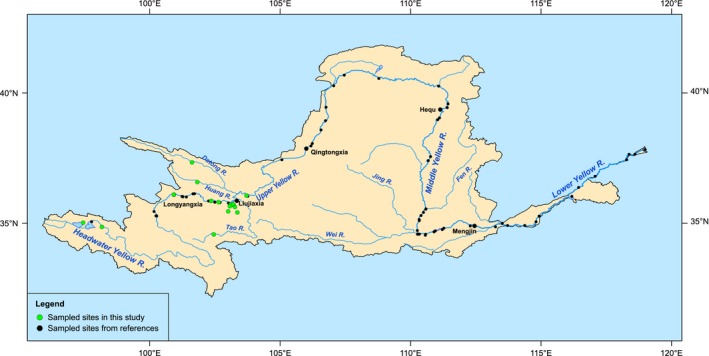
Location of study sites in the Yellow River, China. Green dots denote our sampling sites, and black dots are those from references

We made a detailed use of samples of fish communities originating from two major surveys. One survey defined as a historical data (abbreviation: the 1960s period) was conducted between 1962 and 1963 when only three dams were constructed along the Yellow River (Li, 1965). Li (1965) did not describe his data collection methods in the Yellow River, but they appeared to have been a mixture of samples identified in National Zoological Museum, Li's supplementary data from 1962 to 1963, and the synoptic surveys on fish assemblages collected until 1958 by Institute of Zoology, Chinese Academy of Sciences (Li, 1965). The other one was about 20 years later (CTFRYR, 1986), and we also collected the later fish survey records during 1987–1989 (Chen et al., 1998; Wang, 1991; Yang, 1988; Yue et al., 2000; Zhu, 1989, 1995) (all abbreviation: the 1980s period). The CTFRYR (1986) collected fish via seines, gill nets, and angling during April to October each year based on a standard protocol of Freshwater Specialties of National Fisheries Natural Resources Survey and the Distribution of the Fishery (1980). This team also gleaned information from markets, anglers, and commercial fishers. Those surveys mainly provided presence–absence data of fish species. In addition, during 1990–2015, it was the high‐speed period of hydropower development in China, so the set of data were derived from several recent fish survey records during 1990–2015 and our data during 2010–2015 (abbreviation: the 2000s period). These surveys had the relatively widespread coverage throughout the Yellow River basin (Figure 2; Cai, Zhang, & Wang, 2013; Chen, 2013; Feng, Li, & Zhang, 2009; Li, 2015; Li & Kang, 2012; Liu, Li, Gao, Han, & Zhang, 2010; Liu et al., 2016; Qu, Feng, & Geng, 2011; Ru et al., 2010; Shen, Wang, & Wang, 2013; Tang & He, 2015; Tang, Wang, & Li, 2005; Wang, Li, Hou, & Wen, 2015; Xu, 2011; Zhan, Feng et al., 2009; Zhu, 1999; Zhu, Zhao, Hu, Li, & Wang, 2014). The native and alien species in those data were defined by referring to the boundary of the Yellow River (CTFRYR, 1986; Li & Kang, 2012; Liu et al., 2010; Qu et al., 2011; Tang & He, 2015; Wang, 1991; Xu, 2011). The recent surveys collected fish samples mainly via seines, gill nets, and angling based on two protocols including the one described in the survey published in 1986 (Freshwater specialties of national fisheries natural resources survey and the distribution of fishery, 1980; CTFRYR, 1986; Zhang & He, 1991). Most references only provided presence–absence data of fish species. Based on main sampling sites of the second survey which depended on elevation drop and locations of the large dams in the Yellow River, we selected five reaches from the upper to the lower along the Yellow River mainstem to collect the relevant references from 1965 to 2015. Five segments included the upper reaches of Longyangxia, Longyangxia to Qingtongxia, Qingtongxia to Hequ, Hequ to Mengjin, and the segment down the Mengjin to Bohai Gulf. Moreover, we also collected at 15 sampling points from June to August during 2010–2015 along the upper Yellow River from the Gyaring Lake (97°27′0″E, 35°2′59.75″N) in Qinghai Province, to Lanzhou (103°42′54.25″E, 36°5′23.75″N) in Gansu Province, China, by a gill net or traditional cast net (Figure 2). Specimens identified easily were measured and released in the field. The unidentifiable individuals were transported to the laboratory to identify. A quantity of 500 individuals representing 21 species was collected, which were also found in the published references from 1990 to 2015, and three of them were exotic species (*Cyprinus carpio, Carassius auratus, and Pseudorasbora parva*). Because those data were obtained from diverse survey records, in order to make effective comparisons among the three consecutive period records of fish species, we scrutinized these databases for the accuracy of site locality and unified synonyms of species based on FishBase (http://www.fishbase.org/) and those faunas (Chen et al., 1998; Yue et al., 2000; Zhu, 1989, 1995). Therefore, three temporal separate data sets (the 1960s, the 1980s, and the 2000s period) for each segment of the five sampling segments in the Yellow River were analyzed.

### 2.3 Data analysis

The program PRIMER 5.2.9 was used for multivariate analysis of the fish community data (Clarke & Gorley, 2001). The Bray–Curtis coefficient was used to generate dissimilarity matrices and to quantify fish assemblage dissimilarity between all possible segment pairs within each time period (Clarke, 1993). We also used Bray–Curtis dissimilarity coefficient for pairwise comparison between any two of three periodic groups within five reaches in a period (interperiod within five reaches). Patterns of fish assemblage structure were analyzed using multivariate nonmetric multidimensional scaling ordination (nMDS) (Clarke, 1993). Stress value <0.1 indicates that a valid configuration has been found (Clarke, 1993). To assess differences in the fish assemblage composition, a crossed two‐way multivariate analysis of similarities (ANOSIM) was used (Clarke, 1993). The ANOSIM statistic *R* was calculated for each interperiod pairwise comparison within five reaches. The statistic *R* usually falls between 0 and 1. *R* values close to 0 indicate no separation among groups, while *R* values close to 1 indicate high separation among groups in community composition (Clarke, 1993). Using SPSS v14.0, linear regression was performed to decide whether a relationship existed between the number of fish species and dams.

## 3 RESULTS

### 3.1 Temporal changes of fish assemblages

Species loss was observed in the Yellow River from 1965 to 2015 (Table 1). The number of fish species decreased from 164 to 106 species over the past 50 years. The number of fishes collected by taxonomic level of Order to Species in the 1960s was significantly reduced in 2015, especially by Order taxonomic level in where decreased was up to 53.3%.

**Table 1:** Statistics in the taxonomic level of fishes in the Yellow River, China, from 1965 to 2015

Period	Number of orders	Number of families	Number of genera	Number of fish species	Number of native/exotic fishes
The 1960s	15	28	92	164	163/1
The 1980s	13	26	83	129	112/17
The 2000s	7	18	63	106	80/26

Obvious temporal changes in species assemblages were found with increased nonendemic species and decreased native fishes in the Yellow River from 1965 to 2015 (Table 1 and Figure 4). The proportions of alien fishes in three periods of the 1960s, the 1980s, and the 2000s were 0.6%, 13.1% and 24.5% of the total species, respectively. The number of native fishes and alien fishes of the three‐period groups was associated with increasing numbers of dams in the Yellow River, respectively, while there were not significant (*y* = −3.043*x* + 150.8, *R* ^2^ = .710, *p* = .36 and *y* = 0.903*x* + 5.032, *R* ^2^ = .683, *p* = .38, respectively). Analysis of assemblage dissimilarity also showed a visible change in fish assemblages in the Yellow River, whereby fish composition generally diverged over time. The interperiod Bray–Curtis dissimilarity indexes of fish assemblages in the Yellow River of the 1960s period & the 1980s period, and the 1960s period & the 2000s period were 31.7 and 36.3, respectively. On the other hand, two interperiod indexes of assemblage dissimilarity increased when invasive species were both removed from the analyses (the former and the latter were 34.4 and 39.2, respectively).

**Figure 3:**
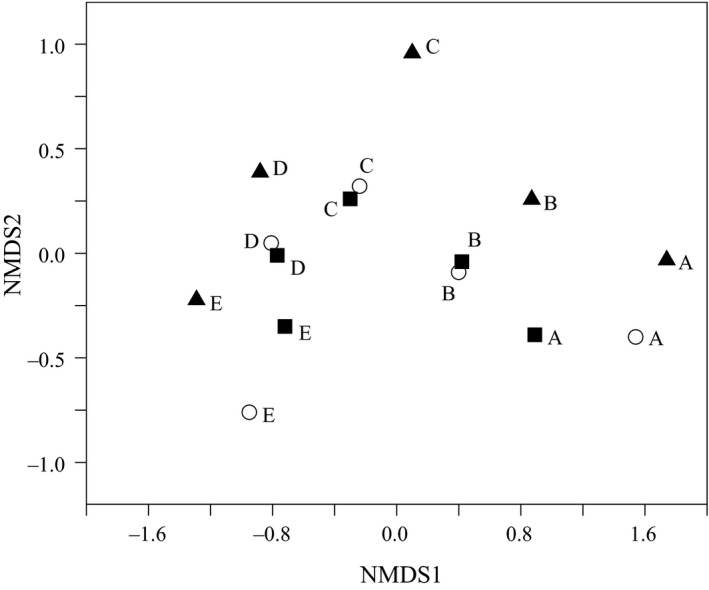
Nonmetric multi dimensional scaling (MDS) based on Bray–Curtis similarity of fish assemblages among five segments in the Yellow River, China, 1965 to 2015. Different geometries denote the sampling periods, closed triangles (▲the 1960s), open circles (○ the 1980s) and closed squares (■ the 2000s). Upper‐case letters denote the sampling segments, A: the upper reaches of Longyangxia; B: Longyangxia to Qingtongxia; C: Qingtongxia to Hequ; D: Hequ to Mengjin; E: the segment downstream of Mengjin

The ANOSIM analysis performed with the total sample subdivided into three‐period groups (the 1960s, the 1980s, and the 2000s period) showed that the Bray–Curtis dissimilarity between paired period in the Yellow River increased with the time intervals, and *R* value indicated that the fish composition was different between the two periods although there was not significant interperiod variability (Table 2, ANOSIM Global *R* = .12, *p* = .17). The Bray–Curtis dissimilarity of each reach in the river also resulted in similar trends using nMDS analysis, and the highest dissimilarity was between the 1960s and the 2000s period (stress = 0.06, Figure 3). Furthermore, the mean Bray–Curtis dissimilarity between paired reaches in the Yellow River within a period decreased from 72.0 to 53.1, although there was no significant interperiod variability of species composition within reaches from 1965 to 2015 (Table 2, ANOSIM Global *R* = .008, *p* = .43).

**Table 2:** Results from the ANOSIM showing all pairwise differences of five reaches in the Yellow River, China, from 1965 to 2015. Mean Bray–Curtis dissimilarity for interperiod within five reaches below the diagonal is compared, and mean Bray–Curtis dissimilarity for inter‐reach within a period in the whole river is along the diagonal

Period	The 1960s	The 1980s	The 2000s
The 1960s	72.0		
The 1980s	67.2	67.2	
The 2000s	66.5	56.0	53.1

### 3.2 Fish assemblage structures varied with reaches over time

**Figure 4:**
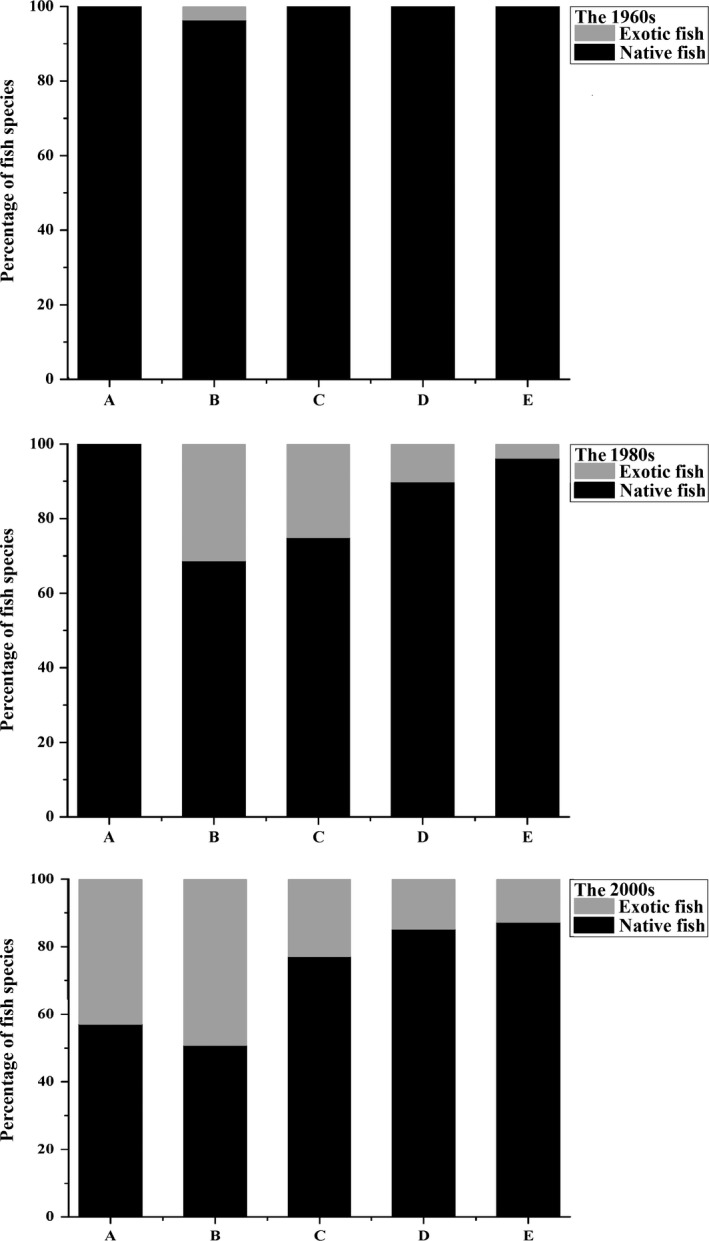
Species richness of fish species assemblages of five segments in the Yellow River, China, 1965–2015, including alien fishes and native fishes. Upper‐case letters denote the sampling segments, A: the upper reaches of Longyangxia; B: Longyangxia to Qingtongxia; C: Qingtongxia to Hequ; D: Hequ to Mengjin; E: the segment downstream of Mengjin

Temporal patterns of species richness and compositions varied with sections from the upper to the lower along the Yellow River (Figure 4). In the upper reaches of Longyangxia, species richness increased from 1965 to 2015 (Figure 4). The composition of fish fauna changed obviously with more alien fishes and fewer natives, while a mean dissimilarity between paired period is 38.8. Only native fishes were caught prior to 1990, whereas 15 exotic fishes were found from 1990 to 2015, representing 42.9% of the total, including stocked species (e.g., *Oncorhynchus mykiss*) and cosmopolitan species (e.g., *Cyprinus carpio*).

The consistent temporal trend in increasing species richness of fishes and the compositional changes were observed in the segment from Longyangxia to Qingtongxia (Figure 4). In the 2000s period, nonindigenous species, such as stocked coldwater Salmonidae fishes, explained 49.1% of the total number compared with 3.6% in the 1960s period, whereas native fishes decreased from 96.4% (the 1960s period) to 50.9% (the 2000s period). Some local lotic species (e.g., *Gobio rivuloides*) and endemic species with spawning migration characteristics (e.g., *Coreius septentrionalis*) were absent in the 2000s period.

In the segment from Qingtongxia to Hequ, species richness increased slightly over time (Figure 4). Increased species richness was mainly due to non‐native species, which accounted for 25.0% and 22.9% of the total in the 1980s period and the 2000s period, respectively. On the other hand, only native fishes were collected prior to 1965. Some local lotic species (e.g., *Acanthogobio guentheri*) and migratory fishes (e.g., *C. septentrionalis*) were absent in the 2000s period.

In the segment from Hequ to Mengjin, species richness decreased slightly over time (Figure 4). Some native fishes decreased or disappeared, such as migratory fishes *Coreius septentrionalis* and *Anguilla japonica*, while introduced fishes increased by five species in the 1980s period.

However, a significant decline in species occurrence was noted at the Mengjin segment, where percent species richness in the 1980s period and the 2000s period was only 61.1% and 35.9% compared to the 1960s period, respectively (Figure 4). Furthermore, fish assemblages in this segment exhibited a distinct change with a mean dissimilarity of 45.0 over time.

## 4 DISCUSSION

Comparing historical and contemporary data sets is necessary both to document patterns of assemblage change and to understand specific ecological responses to environmental alterations (Humphries & Winemiller, 2009). Studies that test for temporal changes in fish assemblages over decades have identified variations (Argent & Carline, 2004; Jacquemin & Pyron, 2011; Penczak, Głowacki, Galicka, & Koszaliński, 1998). Our results also found temporal alterations in fish assemblages in the Yellow River from 1965 to 2015. Up to 35% of fish species in the river were not detected over the past half century, and the rate of species loss accelerated. Moreover, the current fish assemblage of the river consisted of more nonendemic fishes and fewer native fishes compared with the historical composition. The compositions of the fish fauna between reaches in the whole river became more similar over time. Both human influences (e.g., construction dams) and biotic interactions (e.g., biological invasions) are important factors driving riverine fish fauna structure (Johnson, Olden, & Vander Zanden, 2008; Poff et al., 2007‎). The relative importance of these factors probably relies on the perturbation regime of the reach in the river (Poff & Allan, 1995).

In the Yellow River, patterns of temporal changes in fish assemblages varied with reaches from 1965 to 2015. These reaches might experience various types and levels of stressors from anthropogenic perturbations as well as biotic interactions (Gido, Dodds, & Eberle, 2010; Johnson et al., 2008). In the upper reaches of Qingtongxia, the current species composition shifted compared with the historical assemblages from the 1965, exhibiting increased species richness with more invasive species and fewer natives over time. Those that covary with dams and exotic fish invasions could play an important role in structuring temporal fish assemblages in the upper Yellow River and other rivers (Clavero & Garcĭa‐Berthou, 2006; Liermann et al., 2012; Poff et al., 2007).

The development of cascade hydropower exerted a major influence on the current species composition in the upper reaches of the Yellow River at the temporal scale. Habitat alteration through continued dam constructions is regarded as the primary driver in the decline or extirpation of endemic headwater species in other rivers (Gillette et al., 2012; Miller et al., 1989; Tilman, May, Lehman, & Nowak, 1994). These alterations due to the construction and operation of large dams have changed many riverine habitats and fragmented remaining aquatic habitats, and resulted in isolated freshwater fish populations in patches of streams, which may impede organism migration, and thus cause significant threats to native aquatic biodiversity on the globe (Power, Dietrich, & Finlay, 1996). In the Yellow River, based on the historical surveys, twelve of nineteen fish species endemic to the Yellow River inhabited the upper river (Chen et al., 1998; Li, 1965; Zhu, 1989). However, as the earliest hydraulic resources development base in China, 24 of 32 large‐ and medium‐size dams have been constructed in the upper reaches of Qingtongxia since 1961. Nine of twelve endemic species in the upper river are currently listed in the “China Species Red List” (Wang & Xie, 2004). The construction of a cascade hydropower station in the upstream resulted in a continual section of the river divided into a series of pools. In these lentic environments or barriers owing to dam constructions, small‐bodied fishes typical of lotic creek environments fare poorly, but lentic fishes increase, such as *Triplophysa scleroptera* in Longyangxia reservoir (Tang & He, 2015). Meanwhile, diadromous fishes, such as *Gymnodiptychus pachycheilus*, were blocked in the downstream of the river by dams after 1965 (CTFRYR, 1986).

The biological invasion is another important contributor to structuring current fish assemblages by displacing the natives in the upper Yellow River and other freshwater ecosystems (Johnson et al., 2008; Tang & He, 2015). In the upper Yellow River, fish assemblage composition shifted abruptly after 1965, and this shift was mainly due to the occurrence of invasive species. Furthermore, the construction of dams cannot only enhance invasion risk by promoting the establishment and spread of non‐native species (Johnson et al., 2008), but also promote dominance of non‐native species and displace natives through competition and predation (Rahel, 2000). The natural fluxes on continental to global scales effectively prevent the establishment of alien species, while the extensive construction of dams by humans has weakened those fluxes (Poff et al., 2007).

Moreover, aquaculture, the main mechanism of artificial exotic fish introductions, apparently changed current fish assemblages in the upper Yellow River, although it was a by‐product by dams building. Dam constructions provide great breeding conditions for aquaculture or recreational fisheries at global regions (Radomski & Goeman, 1995; Rahel, 2000). Artificial fish introductions also change the reservoirs constructed by the cascade dam into “huge ponds.” The increase in species richness in the upper reaches of the Yellow River was mainly because of many species introductions. Since the 1980s, more species were introduced by stocking into Liujiaxia and Longyangxia reservoirs for economic benefits (Liu, 1984; ‎). For instance, in 1982, those stocked exotic fish accounted for 88.9% of the catch weight (Liu, 1984; ‎). Those exotic fish have become the important aquaculture species in local reservoirs, and the number of introduced species exceeded the number of extirpated native fishes, as elsewhere (Gido & Brown, 1999; Gillette et al., 2012; Tang & He, 2015‎).

In contrast to the upper river, the fish assemblages in the reaches downstream of Hequ experienced significant species loss with additions of a few non‐native species from 1965 to 2015. In particular, in the segment downstream of Mengjin, its species richness reduced by almost two‐third over the past half century. The combined influence of various factors is likely to cause the extensive decline of the fish richness in the downstream.

The flow reduction could be a primary contributor to shaping current fish assemblages in the reaches downstream of Hequ. The middle reaches of the Yellow River pass through the Loess Plateau with low vegetation coverage and high river evaporation. Cessation of downstream flows in summer continuously occurred in the Yellow River during 1972–1998, while such disconnection in the river arrived ahead of time and its period extended every year since the 90s (Liu & Cheng, 2000). The interruptions fundamentally alter the hydrology of the river which fish depended upon. Furthermore, the social and economic consumption of water increased pressures on water scarcity, which diverted about 80% of annual natural flows from the Yellow River since 1997 (Zhan, Zhang et al., 2009). About half of the consumption water is devoted to intensive agricultural uses in the upper‐middle reaches of the Yellow River basin during the fish breeding season, particularly in the Hetao Plain, an important irrigation plain in China (Ru et al., 2010). The diversion of water has resulted in dramatic reductions in base flows, and the extreme minimum freshwater discharge was even only 19.6 m 3/s in 1981, whereby the basic surviving condition has not been maintained for fish (CTFRYR, 1986). Diminished large flows significantly disrupt aquatic life cycles (Scheidegger & Bain, 1995; ‎) and their fish assemblages face intensified biotic interactions and abiotic pressures (Magoulick, 2000).

The dam construction was another important factor responsible for changing downstream fish assemblages of the Yellow River. Dams have the same roles in structuring fish assemblages as in the upper reaches, which cause significant threats and even extinctions of native species (Liermann et al., 2012; Power et al., 1996). Moreover, increased deposition of fine sediments by reservoirs reduces the survival of benthic‐spawning fishes (CTFRYR, 1986). Meanwhile, the construction of dams could also promote dominance of non‐native species (Rahel, 2000), but the disappearance of species in the downstream of the river was not offset by increasing in invasive species over the past half century.

Apart from temporal alteration in fish assemblages, in the Yellow River, the species composition between reaches also became more similar from 1965 to 2015. Homogenization of aquatic biotas has been a worldwide phenomenon (Clavero & Hermoso, 2011; Moyle & Mount, 2007), which is mainly driven by the extirpation of local endemic species and spread of cosmopolitan species (McKinney & Lockwood, 1999). In the Yellow River, this increased similarity is promoting by constructions of dams and introductions of exotic species as in other rivers (Clavero & Garcĭa‐Berthou, 2006; Clavero & Hermoso, 2011; Poff et al., 2007). The construction of dams promotes the decline or extirpation of endemic species (Gillette et al., 2012; Miller et al., 1989; Tilman et al., 1994). By strongly modifying natural flow regimes or being barriers, dams create an enormous amount of more similar lentic freshwater habitat than running water (Poff et al., 2007). Diversified lotic species gradually disappear after habitat homogenization with dam constructions (Clavero & Hermoso, 2011; Moyle & Mount, 2007). Moreover, Dams not only facilitate non‐native species invasions (Poff et al., 2007; Virbickas, Stakėnas, & Steponėnas, 2015), but also are stepping stones to promote colonization of new habitats (Johnson et al., 2008), which might facilitate the future species homogenization.

The invasion of alien species is another important contributor for taxonomic homogenization in the Yellow River (Johnson et al., 2008). Taxonomic homogenization of fish faunas largely attributes to the introductions of the aquaculture and recreation fish at multiple regions (Radomski & Goeman, 1995; Rahel, 2000). When invasive fishes were removed from the analyses, the assemblage dissimilarity between the contemporary and the historical fish assemblages increased in the Yellow River. Over the last 30 years, a large proportion of fish introductions, recorded in the upper and middle Yellow River, were also related to those intentional or accidental stockings (Liu, 1984; Tang & He, 2015‎). Reservoirs dominated by the same suite of introduced fishes replace diverse riverine fauna (Clavero & Hermoso, 2011; Rahel, 2000). The continuous introductions of stocked species and their intrabasin spreads are also producing an apparent decrease in the taxonomic distinctiveness of other basins as in the Yellow River (Antognazza, Andreou, Zaccara, & Britton, 2016; Clavero & Garcĭa‐Berthou, 2006).

## 5 CONCLUSIONS

In conclusion, as anthropogenic environmental alterations and human‐assisted dispersal of exotic species have sparked widespread changes in the distribution of biota at global scales, species assemblages of the Yellow River basin also face decreased fish species richness, increased exotic species, and increased fish species similarity. However, these large‐scale exploited patterns as in the Yellow River have been simulated worldwide. With the rapid economic and urban development in the globe, it is inevitable to exploit large rivers by constructing dams to respond to people's needs (Liermann et al., 2012). Therefore, conservation environments of these rivers may become more critical when population growth and climate change increase the demands placed on freshwater ecosystems (Palmer et al., 2004). Maintaining dynamic regional distinctiveness should be recognized as a conservation priority for the river ecosystem function and indigenous biodiversity (Poff et al., 2007; Western, 2001).Quantitative monitoring of temporal patterns of fish assemblages and the assessment of cumulative effects of cascade hydropower stations on organisms are an important step for understanding responses to riverine alterations. Such knowledge could assist us to evaluate the impact of changes caused by human alteration on the functioning of freshwater ecosystems and to project future changes to the systems (Gido et al., 2010).

## CONFLICT OF INTEREST

The authors declare no conflict of interest.

## AUTHOR CONTRIBUTIONS

Jia Yan Xie designed and directed the study, carried out the data analysis, and wrote the manuscript. Jia Yan Xie, Wen Jia Tang and Yu Hui Yang collected samples, analyzed the data, and contributed to the final writing of the manuscript.

## References

[ece33890-bib-0001] Antognazza, C. M. , Andreou, D. , Zaccara, S. , & Britton, R. J. (2016). Loss of genetic integrity and biological invasions result from stocking and introductions of Barbus barbus: Insights from rivers in England. Ecology and Evolution, 6, 1280–1292. https://doi.org/10.1002/ece3.1906 26843923 10.1002/ece3.1906 PMC4729780

[ece33890-bib-0002] Argent, D. G. , & Carline, R. F. (2004). Fish assemblage changes in relation to watershed landuse disturbance. Aquatic Ecosystem Health & Management, 7, 101–114. https://doi.org/10.1080/14634980490281407

[ece33890-bib-0003] Cai, W. X. , Zhang, J. J. , & Wang, S. W. (2013). Fishes of the Yellow River basin. Yangling, China: Northwest A & F University Press.

[ece33890-bib-0004] Chen, Y. (2013). The biodiversity of fish and protection measures of Yumenkou to Sanmenxia in mainstream of Yellow River. Journal of Anhui Agricultural Sciences, 41, 21 https://doi.org/10.3969/j.issn.0517-6611.2013.15.056

[ece33890-bib-0005] Chen, Y. Y. , Chen, Y. F. , & Liu, H. Z. (1996). Studies on the position of the Qinghai‐Xizang plateau region in zoogeographic divisions and its eastern demarcation line. Acta Hydrobiologica Sinica, 20, 97–103.

[ece33890-bib-0006] Chen, Y. Y. , Chu, X. L. , Luo, Y. L. , Chen, Y. R. , Liu, H. Z. , He, M. J. , … Wu, B. R. (1998). Fauna sinica, osteichthyes cypriniformes II. Beijing, China: Science Press.

[ece33890-bib-0007] Clarke, K. R. (1993). Non‐parametric multivariate analyses of changes in community structure. Australian Journal of Ecology, 18, 117–143. https://doi.org/10.1111/j.1442-9993.1993.tb00438.x

[ece33890-bib-0008] Clarke, K. R. , & Gorley, R. N. (2001). PRIMER v5. User manual/tutorial. Plymouth, UK: PRIMER‐E Ltd.

[ece33890-bib-0009] Clavero, M. , & Garcĭa‐Berthou, E. (2006). Homogenization dynamics and introduction routes of invasive freshwater fish in the Iberian Peninsula. Ecological Applications, 16, 2313–2324. https://doi.org/10.1890/1051-0761(2006)016[2313:hdairo]2.0.co;2 17205906 10.1890/1051-0761(2006)016[2313:hdairo]2.0.co;2

[ece33890-bib-0010] Clavero, M. , & Hermoso, V. (2011). Reservoirs promote the taxonomic homogenization of fish communities within river basins. Biodiversity and Conservation, 20, 41–57. https://doi.org/10.1007/s10531-010-9945-3

[ece33890-bib-0011] CTFRYR (The cooperative team of fishery resource research on the Yellow River system) (1986). Fishery resource in the Yellow River system. Liao Ning, China: Liaoning Science and Technology Publishing House.

[ece33890-bib-0012] Ding, X. , & Zhang, H. (2014). Design of prestressed pier of flood gate, banduo hydropower project. Northwest Water Power, 4, 33–36. https://doi.org/10.3969/j.issn.1006-2610.2014.04.009

[ece33890-bib-0013] Feng, H. , Li, H. , & Zhang, J. (2009). Selection of stocking species in the Yellow River basin. Chinese Fisheries Economics, 27, 85–94. https://doi.org/10.3969/j.issn.1009-590X.2009.05.015

[ece33890-bib-0014] Freshwater Specialties of National Fisheries Natural Resources Survey and the Distribution of the Fishery (1980). Guidelines for the investigation of natural fisheries resources in inland waters. Beijing, China: Freshwater Specialties of National Fisheries Natural Resources Survey and the Distribution of Fishery.

[ece33890-bib-0015] Gido, K. B. , & Brown, J. H. (1999). Invasion of North American drainages by alien fish species. Freshwater Biology, 42, 387–399. https://doi.org/10.1046/j.1365-2427.1999.444490.x

[ece33890-bib-0016] Gido, K. B. , Dodds, W. K. , & Eberle, M. E. (2010). Retrospective analysis of fish community change during a half‐century of landuse and streamflow changes. Journal of the North American Benthological Society, 29, 970–987. https://doi.org/10.1899/09-116.1

[ece33890-bib-0017] Gillette, D. P. , Fortner, A. M. , Franssen, N. R. , Cartwright, S. , Tobler, C. M. , Wesner, J. S. , … Lee, C. W. (2012). Patterns of change over time in darter (Teleostei: Percidae) assemblages of the Arkansas River basin, Northeastern Oklahoma, USA. Ecography, 35, 855–864. https://doi.org/10.1111/j.1600-0587.2011.06560.x

[ece33890-bib-0018] Głowacki, Ł. B. , & Penczak, T. (2013). Drivers of fish diversity, homogenization/differentiation and species range expansions at the watershed scale. Diversity and Distributions, 19, 907–918. https://doi.org/10.2307/23479811

[ece33890-bib-0019] Graf, W. L. (2006). Downstream hydrologic and geomorphic effects of large dams on American rivers. Geomorphology, 79, 336–360. https://doi.org/10.1016/j.geomorph.2006.06.022

[ece33890-bib-0020] He, D. , & Chen, Y. (2006). Biogeography and molecular phylogeny of the genus schizothorax (Teleostei, Cyprinidae) in China inferred from cytochrome b sequences. Journal of Biogeography, 33, 1448–1460. https://doi.org/10.1111/j.1365-2699.2006.01510.x

[ece33890-bib-0021] Hooper, D. U. , Chapin, F. S. , Ewel, J. J. , Hector, A. , Inchausti, P. , Lavorel, S. , … Wardle, D. A. (2005). Effects of biodiversity on ecosystem functioning, a consensus of current knowledge. Ecological Monographs, 75, 3–35. https://doi.org/10.3410/f.1026316.367900

[ece33890-bib-0022] Humphries, P. , & Winemiller, K. O. (2009). Historical impacts on river fauna, shifting baselines, and challenges for restoration. BioScience, 59, 673–684. https://doi.org/10.1525/bio.2009.59.8.9

[ece33890-bib-0023] Jacquemin, S. J. , & Pyron, M. (2011). Fishes of Indiana streams: Current and historic assemblage structure. Hydrobiologia, 665, 39–50. https://doi.org/10.1007/s10750-011-0602-y

[ece33890-bib-0024] Johnson, P. T. J. , Olden, J. D. , & Vander Zanden, M. J. (2008). Dam invaders, impoundments facilitate biological invasions into freshwaters. Frontiers in Ecology and the Environment, 6, 357–363. https://doi.org/10.1890/070156

[ece33890-bib-0025] Kou, X. , Niu, T. , Huang, Y. , Pang, W. , & Yang, Z. (2009). Cumulative effect of water environment of existing cascade stations on the upper Yellow River. Northwest Hydropower, 6, 11–14. https://doi.org/10.3969/j.issn.1006-2610.2009.06.003

[ece33890-bib-0026] Lehner, B. , Liermann, C. R. , Revenga, C. , Vörösmarty, C. , Fekete, B. , Crouzet, P. , … Wisser, D. (2011). High‐resolution mapping of the world's reservoirs and dams for sustainable river‐flow management. Frontiers in Ecology and the Environment, 9, 494–502. https://doi.org/10.1890/100125

[ece33890-bib-0027] Li, S. Z. (1965). Research on the ichthyofauna in the Yellow River. Chinese Journal of Zoology, 5, 217–222.

[ece33890-bib-0028] Li, W. (2005). Hydroelectric exploitation on the main stream of the Yellow River Gansu province and regional economic development. Water Power, 31, 1–5. https://doi.org/10.3969/j.issn.0559-9342.2005.04.001

[ece33890-bib-0029] Li, S. Z. (2015). Fishes of the Yellow River and beyond. Keelung, Taiwan: The Sueichan Press.

[ece33890-bib-0030] Li, Q. , & Kang, P. (2012). General situation of aboriginal fish resources and utilization and protection Strategy in Gansu province. Freshwater Fisheries, 42, 92–96. https://doi.org/10.3969/j.issn.1000-6907.2012.03.019

[ece33890-bib-0031] Liermann, C. R. , Nilsson, C. , Robertson, J. , & Ng, R. Y. (2012). Implications of dam obstruction for global freshwater fish diversity. BioScience, 62, 539–548. https://doi.org/10.1525/bio.2012.62.6.5

[ece33890-bib-0032] Liu, Y. G. (1984). Investigation on the fishery status and yield of the Liujiaxia reservoir. Gansu Agricultural Science and Technology, 10, 1–5.

[ece33890-bib-0033] Liu, C. , & Cheng, L. (2000). Analysis on runoff series with special reference to drying up courses of lower Huanghe River. Acta Geographica Sinica, 55, 257–265. https://doi.org/10.3321/j.issn:0375-5444.2000.03.001

[ece33890-bib-0034] Liu, H. , Du, Z. , An, X. , Li, Z. , An, M. , Wu, E. , & Wulantuoya, W. (2016). Fish fauna of the Yellow River in Inner Mongolia reaches and the new findings. Journal of Northern Agriculture, 44, 86–90. https://doi.org/10.3969/j.issn.2096-1197.2016.01.21

[ece33890-bib-0035] Liu, X. , Li, K. , Gao, H. , Han, J. , & Zhang, J. (2010). Research and protective strategy of the fishery resource in Ningxia‐Neimeng River reach of Yellow River trunk stream. Journal of Hydroecology, 3, 135–141.

[ece33890-bib-0036] Magoulick, D. D. (2000). Spatial and temporal variation in fish assemblages of drying stream pools, the role of abiotic and biotic factors. Aquatic Ecology, 34, 29–41. https://doi.org/10.1023/A:1009914619061

[ece33890-bib-0037] McKinney, M. L. , & Lockwood, J. L. (1999). Biotic homogenization: A few winners replacing many losers in the next mass extinction. Trends in Ecology & Evolution, 14, 450–453. https://doi.org/10.1016/s0169-5347(99)01679-1 10511724 10.1016/s0169-5347(99)01679-1

[ece33890-bib-0038] Miller, R. R. , Williams, J. D. , & Williams, J. E. (1989). Extinctions of North American fishes during the past century. Fisheries, 14, 22–36. https://doi.org/10.1577/1548-8446(1989)014%3C0022:eonafd%3E2.0.co;2

[ece33890-bib-0039] Mims, M. C. , & Olden, J. D. (2013). Fish assemblages respond to altered flow regimes via ecological filtering of life history strategies. Freshwater Biology, 58, 50–62. https://doi.org/10.1111/fwb.12037

[ece33890-bib-0040] Moyle, P. B. , & Mount, J. F. (2007). Homogenous rivers, homogenous faunas. Proceedings of the National Academy of Sciences of the United States of America, 104, 5711–5712. https://doi.org/10.1073/pnas.0701457104 17392424 10.1073/pnas.0701457104 PMC1851555

[ece33890-bib-0041] Olden, D. J. , Poff, N. L. , Douglas, M. R. , Douglas, M. E. , & Fausch, K. D. (2004). Ecological and evolutionary consequences of biotic homogenization. Trends in Ecology & Evolution, 19, 18–23. https://doi.org/10.1016/j.tree.2003.09.010 16701221 10.1016/j.tree.2003.09.010

[ece33890-bib-0042] Olden, J. D. , & Rooney, T. P. (2006). On defining and quantifying biotic homogenization. Global Ecology and Biogeography, 15, 113–120. https://doi.org/10.1111/j.1466-822x.2006.00214.x

[ece33890-bib-0043] Palmer, M. , Bernhardt, E. , Chornesky, E. , Collins, S. , Dobson, A. , Duke, C. , … Turner, M. (2004). Ecology for a crowded planet. Science, 304, 1251–1252. https://doi.org/10.1126/science.1095780 15166349 10.1126/science.1095780

[ece33890-bib-0044] Palmer, M. A. , Reidy Liermann, C. A. , Nilsson, C. , Flörke, M. , Alcamo, J. , Lake, P. S. , & Bond, N. (2008). Climate change and world's river basins: Anticipating management options. Frontiers in Ecology and the Environment, 6, 81–89. https://doi.org/10.1890/060148

[ece33890-bib-0045] Penczak, T. , Głowacki, Ł. , Galicka, W. , & Koszaliński, H. (1998). A long‐term study (1985–1995) of fish populations in the impounded Warta river, Poland. Hydrobiologia, 368, 157–173.

[ece33890-bib-0046] Poff, N. L. , & Allan, J. D. (1995). Functional organization of stream fish assemblages in relation to hydrological variability. Ecology, 76, 606–627. https://doi.org/10.2307/1941217

[ece33890-bib-0047] Poff, N. L. , Olden, J. D. , Merritt, D. , & Pepin, D. (2007). Homogenization of regional river dynamics by dams and global biodiversity implications. Proceedings of the National Academy of Sciences of the United States of America, 104, 5732–5737. https://doi.org/10.1073/pnas.0609812104 17360379 10.1073/pnas.0609812104 PMC1851560

[ece33890-bib-0048] Poff, N. L. , & Zimmerman, J. K. H. (2010). Ecological responses to altered flow regimes: A literature review to inform the science and management of environmental flows. Freshwater Biology, 55, 194–205. https://doi.org/10.1111/j.1365-2427.2009.02272.x

[ece33890-bib-0049] Power, M. E. , Dietrich, W. E. , & Finlay, J. C. (1996). Dams and downstream aquatic biodiversity, potential food web consequences of hydrologic and geomorphic change. Journal of Environmental Management, 20, 887–895. https://doi.org/10.1007/bf01205969 10.1007/BF01205969 8895411

[ece33890-bib-0050] Qu, C. , Feng, J. , & Geng, R. (2011). Analysis of fauna composition in the Yellow River Basin (Henan section). Henan Fisheries, 4, 32–34.

[ece33890-bib-0051] Radomski, P. J. , & Goeman, T. J. (1995). The homogenizing of Minnesota Lake fish assemblages. Fisheries, 20, 20–23. https://doi.org/10.1577/1548-8446(1995)020%3C0020:thomlf%3E2.0.co;2

[ece33890-bib-0052] Rahel, F. J. (2000). Homogenization of fish faunas across the United States. Science, 288, 854–856. https://doi.org/10.1126/science.288.5467.854 10797007 10.1126/science.288.5467.854

[ece33890-bib-0053] Ru, H. , Wang, H. , Zhao, W. , Shen, Y. , Wang, Y. , & Zhang, X. (2010). Fishes in the mainstream of the Yellow River, assemblage characteristics and historical changes. Biodiversity Science, 18, 169–174. https://doi.org/10.3724/SP.J.1003.2010.179

[ece33890-bib-0054] Scheidegger, K. J. , & Bain, M. B. (1995). Larval fish distribution and microhabitat use in free flowing and regulated rivers. Copeia, 1, 125–135. https://doi.org/10.2307/1446807

[ece33890-bib-0055] Shen, Z. , Wang, G. , & Wang, Z. (2013). Evaluation of fish culture and propagation of white salmon in Qinghai Province. Fisheries of China, 12, 62–64.

[ece33890-bib-0056] Sun, W. , Zhuang, D. , Zhang, S. , & Zheng, H. (2012). Analysis on dam seepage safety of Huangheyuan hydropower station. Hydro‐Science and Engineering, 1, 62–70. https://doi.org/10.3969/j.issn.1009-640X.2012.01.011

[ece33890-bib-0057] Tang, W. , & He, D. (2013). Fish resource survey on Cihaxia to Jishixia stretches in the upper reaches of Yellow River (2005–2010). Lake Science, 25, 600–608. https://doi.org/10.18307/2013.0419

[ece33890-bib-0058] Tang, W. , & He, D. (2015). Investigation on alien fishes in Qinghai province of China, from 2001 to 2014. Journal of Lake Sciences, 27, 502–510. https://doi.org/10.18307/2015.0318

[ece33890-bib-0059] Tang, W. , Wang, M. , & Li, K. (2005). Indigen fish in Qinghai province. Chinese Journal of Fisheries, 18, 13–17. https://doi.org/10.3969/j.issn.1005-3832.2005.01.003

[ece33890-bib-0060] The Yellow River Conservancy Commission of Ministry of Water Resources Water (2012). Resources bulletin of the Yellow River in 2012. Retrieved from http://www.yellowriver.gov.cn/other/hhgb/

[ece33890-bib-0061] Tilman, D. , May, R. M. , Lehman, C. L. , & Nowak, M. A. (1994). Habitat destruction and the extinction debt. Nature, 371, 65–66. https://doi.org/10.1038/371065a0

[ece33890-bib-0062] Virbickas, T. , Stakėnas, S. , & Steponėnas, A. (2015). Impact of beaver dams on abundance and distribution of anadromous salmonids in two lowland streams in Lithuania. PLoS ONE, 10, e0123107 https://doi.org/10.1371/journal.pone.0123107 25856377 10.1371/journal.pone.0123107 PMC4391911

[ece33890-bib-0063] Wang, X. T. (1991). Vertebrate fauna of Gansu. Lanzhou, China: Gansu Science and Technology Press.

[ece33890-bib-0064] Wang, Y. , Li, W. , Hou, S. , & Wen, S. (2015). Investigation on fishery resources in Shaanxi section of the Yellow River. Freshwater Fisheries, 2, 97–101. https://doi.org/10.3969/j.issn.1000-6907.2015.02.017

[ece33890-bib-0065] Wang, S. , & Xie, Y. (2004). China species red list. Beijing, China: Higher Education Press.

[ece33890-bib-0066] Western, D. (2001). Human‐modified ecosystems and future evolution. Proceedings of the National Academy of Sciences of the United States of America, 98, 5458–5465. https://doi.org/10.1073/pnas.101093598 11344294 10.1073/pnas.101093598 PMC33234

[ece33890-bib-0067] Xu, R. (2011). Fauna inner Mongolia (volume I cyclostomata and pisces). Hohhot, China: Inner Mongolia University Press.

[ece33890-bib-0068] Yang, M. (1988). Supplementary records of indigenous fish in Ningxia. Ningxia Journal of Agriculture and Forestry Science and Technology, 4, 50–51.

[ece33890-bib-0069] Yang, X. (1997). Issues having to be pay attention in development of the key projects in the main Yellow River stem. Yellow River, 6, 49–52.

[ece33890-bib-0070] Yang, Q. , & Xin, S. (1998). Achievements and effect analysis of hydropower construction in the Yellow River. Yellow River, 20, 26–28.

[ece33890-bib-0071] Yue, P. Q. , Shan, X. H. , Lin, R. D. , Chu, X. L. , Zhang, E. , Chen, J. X. , … Wu, B. L. (2000). Fauna sinica (osteichthyes cypriniformes III). Beijing, China: Science Press.

[ece33890-bib-0072] Zhan, J. , Feng, H. , Li, K. , Yang, X. , Li, F. , & Zhang, J. (2009). Changes of fishery resources after the construction of cascade hydropower stations from Longyang Gorge to Liujia Gorge in upper stream of Yellow River. Freshwater Fisheries, 39, 40–45. https://doi.org/10.3969/j.issn.1000-6907.2009.03.008

[ece33890-bib-0073] Zhan, Q. , Zhang, J. , & Qing, C. (2009). Characteristics of installation and techniques of water‐turbine generator sets of Xiayuan hydroelectric plant. Yellow River, 10, 27–28. https://doi.org/10.3969%2fj.issn.1000-1379.2009.10.014

[ece33890-bib-0074] Zhang, J. , & He, Z. (1991). Handbook for the investigation of natural fisheries resources in inland waters. Beijing, China: Agricultural Press.

[ece33890-bib-0075] Zhu, S. Q. (1989). The loaches of the subfamily nemachelinae in China (Cypriniformes, Cobitidae). Nanjing, China: Jiangsu Science and Technology Publishing House.

[ece33890-bib-0076] Zhu, S. Q. (1995). Synopsis of freshwater fishes of China. Nanjing, China: Jiangsu Science and Technology Publishing House.

[ece33890-bib-0077] Zhu, Y. (1999). Idea on cascade development of hydroelectric resources of dabeiganliu main upstream of the Yellow River. Shanxi Hydrotechnics, 2, 38–40.

[ece33890-bib-0078] Zhu, G. , Zhao, R. , Hu, Z. , Li, L. , & Wang, X. (2014). Fish Distribution and species diversity in major rivers in Shanxi Province. Chinese Journal of Fisheries, 2, 38–45. https://doi.org/10.3969/j.issn.1005-3832.2014.02.008

