# Supplementary material for: Fish assemblage changes over half a century in the Yellow River, China
Source: Ecol Evol. 2018 Mar 30;8(8):4173–82. doi: 10.1002/ece3.3890 (PMC5916296; doi:10.1002/ece3.3890)
Supplement: Supplementary file 1 [file ECE3-8-4173-s001.docx]

**Fish assemblage changes over half a century in the Yellow River, China**

**Jia Yan Xie ^1,*^, Wen Jia Tang^2^, Yu Hui Yang^1^**

^1^ School of Biology and Pharmaceutical Engineering, Wuhan Polytechnic University, Wuhan 430023, Hubei, China,

^2^ Qinghai Eco-environment Remote Sensing Monitoring Center, Xining 810000, China

**^*^** Correspondence: [xjyaphid@163.com](mailto:xjyaphid@163.com); Tel.: + 86-27-83956793

# Supporting Information

### Appendix 1 Fish composition in the Yellow River, China

| Species | The 1960s | The 1980s | The 2000s |
| --- | --- | --- | --- |
| *Acipenser dabryanus* | + |  |  |
| *Huso dauricus* | + |  |  |
| *Huso gladius* | + |  |  |
| *Setipinna taty* |  | + |  |
| *Coilia ectenes* |  | + |  |
| *Coilia mystus* | + |  |  |
| *Anguilla japonica* | + | + |  |
| *Brachymystax lenok* | + |  |  |
| *Oncorhynchus mykiss* |  | + | + |
| *Salvelinus fontinalis* |  |  | + |
| *Coregonus muksun* |  |  | + |
| *Coregonus nasus* |  |  | + |
| *Coregonus peled* |  |  | + |
| *Coregonus ussuriensis* | + |  |  |
| *Plecoglossus altivelis* | + |  |  |
| *Hypomesus nipponensis* |  |  | + |
| *Neosalanx taihuensis* |  |  | + |
| *Neosalanx anderssoni* | + |  |  |
| *Protosalanx hyalocranius* | + |  | + |
| *Hemisalanx prognathus* |  | + |  |
| *Hemisalanx brachyrostralis* |  | + |  |
| *Salanx ariakensis* | + | + |  |
| *Opsariichthys bidens* | + | + | + |
| *Zacco platypus* | + | + | + |
| *Aphyocypris chinensis* | + | + |  |
| *Mylopharyngodon piceus* | + | + | + |
| *Ctenoparyngodon idellus* | + | + | + |
| *Ochetobius elongatus* | + |  |  |
| *Elopichthys bambusa* | + | + |  |
| *Rhynchocypris lagowskii* | + | + | + |
| *Phoxinus oxycephalus* | + |  |  |
| *Leuciscus waleckii* | + | + | + |
| *Leuciscus chuanchicus* | + | + | + |
| *Squaliobarbus curriculus* | + | + | + |
| *Hypophthalmichthys molitrix* | + | + | + |
| *Aristichthys nobilis* | + | + | + |
| *Pseudolaubuca sinensis* | + |  | + |
| *Pseudolaubuca engraulis* | + | + | + |
| *Toxabramis swinhonis* | + |  |  |
| *Hemiculter leucisculus* | + | + | + |
| *Hemiculter bleekeri* | + | + | + |
| *Cultrichthys erythropterus* | + | + | + |
| *Culter alburnus* | + | + | + |
| *Chanodichthys mongolicus* | + |  | + |
| *Chanodichthys oxycephalus* | + |  |  |
| *Chanodichthys dabryi* | + |  |  |
| *Parabramis pekinensis* | + | + | + |
| *Megalobrama terminalis* | + | + |  |
| *Megalobrama amblycephala* |  | + | + |
| *Xenocypris macrolepis* | + | + | + |
| *Plagiognathops microlepis* | + |  |  |
| *Xenocypris davidi* | + | + |  |
| *Pseudobrama simoni* | + | + | + |
| *Acheilognathus macropterus* | + |  | + |
| *Acheilognathus taenianalis* | + | + |  |
| *Acheilognathus chankaensis* | + | + | + |
| *Acheilognathus barbatulus* | + | + |  |
| *Acheilognathus tonkinensis* |  | + |  |
| *Acheilognathus imberbis* | + |  | + |
| *Rhodeus fangi* | + |  |  |
| *Rhodeus lighti* |  | + | + |
| *Rhodeus ocellatus* | + | + | + |
| *Rhodeus sinensis* | + | + | + |
| *Abbottina rivularis* | + | + | + |
| *Gnathopogon polytaenia* | + |  |  |
| *Gnathopogon imberbis* | + |  |  |
| *Gnathopogon tsinanensis* | + | + |  |
| *Paraleucogobio strigatus* | + |  |  |
| *Paraleucogobio notacanthus* | + |  |  |
| *Gobio soldatovi* | + |  |  |
| *Gobio cynocephalus* | + | + | + |
| *Romanogobio tenuicorpus* | + |  | + |
| *Gobio coriparoides* | + | + | + |
| *Gobio huanghensis* | + | + | + |
| *Gobio rivuloides* | + | + | + |
| *Gobio meridionalis* | + |  |  |
| *Hemibarbus labeo* | + | + | + |
| *Hemibarbu maculatus* | + | + | + |
| *Pseudorasbora parva* | + | + | + |
| *Sarcocheilichthys sinensis* | + | + |  |
| *Sarcocheilichthys nigripinnis* | + | + |  |
| *Saurogobio dabryi* | + | + | + |
| *Saurogobio dumerili* | + | + | + |
| *Saurogobio gymnocheilus* |  | + |  |
| *Squalidus argentatus* | + | + |  |
| *Squalidus intermedius* | + |  | + |
| *Squalidus nitens* |  | + |  |
| *Squalidus wolterstorffi* | + | + |  |
| *Coreius heterodon* | + | + | + |
| *Coreius septentrionalis* | + | + | + |
| *Coreius cetopsis* | + |  |  |
| *Rhinogobio nasutus* | + | + | + |
| *Rhinogobio typus* | + | + |  |
| *Rhinogobio cylindricus* |  | + | + |
| *Huigobio chinssuensis* | + | + | + |
| *Pseudogobio vaillanti* | + | + | + |
| *Acanthogobio guentheri* | + | + | + |
| *Gobiobotia pappenheimi* | + | + | + |
| *Gobiobotia brevirostris* |  | + |  |
| *Gobiobotia homalopteroidea* | + | + | + |
| *Gobiobotia filifer* | + | + | + |
| *Onychostoma macrolepis* | + | + |  |
| *Gymnodiptychus pachycheilus* | + | + | + |
| *Gymnocypris eckloni eckloni* | + | + | + |
| *Gymnocypris eckloni scoliostomus* |  | + | + |
| *Schizopygopsis pylzovi* | + | + | + |
| *Chuanchia labiosa* | + | + | + |
| *Platypharodon extremus* | + | + | + |
| *Cyprinus carpio* | + | + | + |
| *Carassius auratus* | + | + | + |
| *Lefua costata* | + | + |  |
| *Barbatula toni* | + | + |  |
| *Triplophysa sellaefer* | + |  |  |
| *Triplophysa robusta* | + | + | + |
| *Triplophysa heyangensis* | + |  |  |
| *Triplophysa dalaica* | + | + | + |
| *Triplophysa stoliczkai* | + | + | + |
| *Triplophysa leptosoma* | + | + | + |
| *Triplophysa brevicauda* | + |  | + |
| *Triplophysa orientalis* | + | + | + |
| *Triplophysa moquensis* | + |  |  |
| *Triplophysa obscura* | + |  |  |
| *Triplophysa scleroptera* | + | + | + |
| *Triplophysa pseduscleroptera* |  | + | + |
| *Triplophysa pappenheimi* | + | + | + |
| *Triplophysa siluroides* | + | + | + |
| *Triplophysa obtusirostra* | + |  | + |
| *Triplophysa longianguis* | + |  | + |
| *Triplophysa stenura* | + |  |  |
| *Triplophysa alticeps* |  |  | + |
| *Triplophysa crassilabris* | + |  |  |
| *Triplophysa hutjertjuensis* | + |  | + |
| *Triplophysa microps* |  |  | + |
| *Leptobotia orientalis* | + |  |  |
| *Leptobotia elongata* |  |  | + |
| *Parabotia fasciata* | + | + |  |
| *Cobitis sibirica* | + | + | + |
| *Cobitis melanoleuca* | + |  |  |
| *Cobitis sinensis* | + | + | + |
| *Misgurnus mohoity* |  |  | + |
| *Misgurnus anguillicaudatus* | + | + | + |
| *Paramisgurnus dabryanus* | + | + | + |
| *Silurus asotus* | + | + | + |
| *Silurus lanzhouensis* | + | + | + |
| *Clarias fuscus* |  |  | + |
| *Pelteobagrus fulvidraco* | + | + | + |
| *Pelteobagrus vachelli* | + | + | + |
| *Pelteobagrus nitidus* |  | + | + |
| *Tachysurus argentivittatus* | + |  |  |
| *Leiocassis longirostris* | + |  |  |
| *Leiocassis crassilabris* | + | + | + |
| *Pseudobagrus kaifenensis* | + | + |  |
| *Pseudobagrus ussuriensis* | + | + | + |
| *Pseudobagrus ondon* | + |  | + |
| *Pseudobagrus albomarginatus* | + |  |  |
| *Pseudobagrus truncatus* | + |  |  |
| *Siniperca chuatsi* | + |  | + |
| *Siniperca scherzeri* | + |  |  |
| *Lateolabrax japonicus* | + | + |  |
| *Lateolabrax maculatus* | + |  |  |
| *Collichthys lucidus* |  | + |  |
| *Micropercops cinctus* | + | + | + |
| *Nibea albiflora* |  | + |  |
| *Odontobutis obscura* | + | + |  |
| *Rhinogobius cliffordpopei* |  | + | + |
| *Rhinogobius giurinus* | + | + | + |
| *Rhinogobius lindbergi* |  |  | + |
| *Triaenopogon barbatus* |  | + |  |
| *Chaeturichthys stigmatias* | + | + |  |
| *Acanthogobius flavimanus* | + |  |  |
| *Acanthogobius lactipes* |  | + |  |
| *Acanthogobius elongata* | + |  |  |
| *Synechogobius ommaturus* |  | + |  |
| *Acanthogobius hasta* | + |  |  |
| *Tridentiger trigonocephalus* | + | + |  |
| *Tridentiger obscurus* | + |  |  |
| *Lophiogobius ocellicauda* |  | + |  |
| *Odontamblyopus rubicundus* | + | + |  |
| *Glossogobius giuris* | + |  |  |
| *Periophthalmus modestus* | + | + |  |
| *Pamspus argenteus* |  | + |  |
| *Macropodus opercularis* | + | + | + |
| *Channa argus* | + | + | + |
| *Trachidermus fasciatus* | + | + |  |
| *Platycephalus indicus* |  | + |  |
| *Liza haematocheila* | + | + |  |
| *Hemiramphus kurumeus* |  | + |  |
| *Hyporhamphus sajori* | + |  |  |
| *Hyporhamphus intermedius* | + |  |  |
| *Oryzias sinensis* | + | + | + |
| *Pungitius sinensis* | + |  | + |
| *Syngnathus acus* | + |  |  |
| *Monopterus albus* | + | + | + |
| *Mastacembelus aculeatus* | + | + | + |
| *Cleisthenes herzensteini* |  | + |  |
| *Zebrias zebra* |  | + |  |
| *Cynoglossus gracilis* |  | + |  |
| *Cynoglossus trigrammus* |  | + |  |
| *Cynoglossus semilaevis* | + |  |  |
| *Cynoglossus joyneri* | + |  |  |
| *Cynoglossus abbreviatus* | + |  |  |
| *Takifugu ocellatus* | + |  |  |
| *Takifugu obscurus* | + | + |  |
| *Takifugu rubripes* | + |  |  |
| *Takifugu niphobles* | + |  |  |
